# Transcriptome analysis of chrysanthemum (*Dendranthema grandiflorum*) in response to low temperature stress

**DOI:** 10.1186/s12864-018-4706-x

**Published:** 2018-05-02

**Authors:** Ke Wang, Zhen-yu Bai, Qian-yu Liang, Qing-lin Liu, Lei Zhang, Yuan-zhi Pan, Guang-li Liu, Bei-bei Jiang, Fan Zhang, Yin Jia

**Affiliations:** Department of Ornamental Horticulture, Sichuan Agricultural University, 211 Huimin Road, Wenjiang District, Chengdu, Sichuan 611130 People’s Republic of China

**Keywords:** *Dendranthema grandiflorum*, Low temperature stress, Cold acclimation, Transciptome sequence, Differentially expressed genes

## Abstract

**Background:**

Chrysanthemum is one kind of ornamental plant well-known and widely used in the world. However, its quality and production were severely affected by low temperature conditions in winter and early spring periods. Therefore, we used the RNA-Seq platform to perform a *de novo* transcriptome assembly to analyze chrysanthemum (*Dendranthema grandiflorum*) transcription response to low temperature.

**Results:**

Using Illumina sequencing technology, a total of 86,444,237 high-quality clean reads and 93,837 unigenes were generated from four libraries: T01, controls; T02, 4 °C cold acclimation (CA) for 24 h; T03, − 4 °C freezing treatments for 4 h with prior CA; and T04, − 4 °C freezing treatments for 4 h without prior CA. In total, 7583 differentially expressed genes (DEGs) of 36,462 annotated unigenes were identified. We performed GO and KEGG pathway enrichment analyses, and excavated a group of important cold-responsive genes related to low temperature sensing and signal transduction, membrane lipid stability, reactive oxygen species (ROS) scavenging and osmoregulation. These genes encode many key proteins in plant biological processes, such as protein kinases, transcription factors, fatty acid desaturase, lipid-transfer proteins, antifreeze proteins, antioxidase and soluble sugars synthetases. We also verified expression levels of 10 DEGs using quantitative real-time polymerase chain reaction (qRT-PCR). In addition, we performed the determination of physiological indicators of chrysanthemum treated at low temperature, and the results were basically consistent with molecular sequencing results.

**Conclusion:**

In summary, our study presents a genome-wide transcript profile of *Dendranthema grandiflorum* var. jinba and provides insights into the molecular mechanisms of *D. grandiflorum* in response to low temperature. These data contributes to our deeper relevant researches on cold tolerance and further exploring new candidate genes for chilling-tolerance and freezing-tolerance chrysanthemum molecular breeding.

**Electronic supplementary material:**

The online version of this article (10.1186/s12864-018-4706-x) contains supplementary material, which is available to authorized users.

## Background

Low temperature is a common environmental stress factor, which seriously affects plant growth and becomes an important factors limiting plant distribution. Plants will make a series of physiological and biochemical changes as defense systems in response to chilling (0–15 °C) and freezing (< 0 °C) injury [1]. These cold resistance mechanisms involve biofilm system, osmotic regulators, protective enzyme systems, and fatty acids. For many temperate plant species, exposed to chilling temperature for a period of time could increase their tolerance to subsequent freezing conditions, and this process is called ‘cold acclimation (CA)’ [2]. Cold acclimation can regulate many cold-responsive genes differentially expressed, induce the establishment of cold resistance mechanism, and finally improve the adaptability of plants to low temperature [3].

*Dendranthema grandiflorum* var. jinba is one of the best-selling cut chrysanthemum varieties in China. In order to realize the annual production and supply of cut chrysanthemum, in most areas of China, factories need to use the facilities for heating in winter, which greatly increases the cost of production. Thus, understanding the mechanism of chilling and freezing stress responses and improving the cold tolerance of chrysanthemum by gene transfer are of great importance.

Chrysanthemum has large genomes and lacks genomic information on the basis of cold tolerance. With the development of next-generation sequencing (NGS) technology, large scale transcriptome data has become availavle in various species [4]. In recent years, Illumina RNA-Seq technology has been successfully applied to many plant species, such as *Beta vulgaris* [5], *Spartina pectinata* [6], *Camellia sinensis* [7], *Lilium lancifolium* [8], and *Camellia japonica* [9], for its high accuracy and sensitivity of gene discovery.

In our study, based on Illumina NGS technology, a fully characterized chrysanthemum transcriptome was represented. Physiological experiments and molecular sequencing analysis combined to explore the cold mechanism of chrysanthemum under chilling and freezing stresses. Moreover, we compare and analyze the physiological and molecular aspects of chrysanthemum with and without cold acclimation to explore the effect of acclimation on chrysanthemum. Lots of cold-induced genes were identified, and some of them were of great importance in cold-tolerance chrysanthemum molecular breeding.

## Methods

### Plant materials and low temperature treatments

*Dendranthema grandiflorum* var. jinba was used in this study. The buds raised from tissue-cultured seedlings were grown on MS medium (16 h photoperiod, 25 °C/22 °C day/night temperature) for twenty days. Then twenty-day old chrysanthemum seedlings were transferred to pots filled with a 1:1 mixture of peat and perlite, and acclimated for three days at normal condition. Then the four groups of seedlings were respectively subjected to following treatments: (T01) normal condition as control, (T02) 4 °C for 24 h, (T03) 4 °C for 24 h, followed by − 4 °C for 4 h, (T04) -4 °C for 4 h. Each treatment collected a mixture of three biologically replicated leaves, then samples rapidly frozen with liquid nitrogen and stored at − 80 °C. There were four samples in total used for RNA-Seq and differential expression analyses.

### RNA preparation

Total RNA was extracted according to manufacturer’s instructions. 1% agarose gels was used for the monitoring of RNA degradation and contamination; the NanoPhotometer® spectrophotometer (IMPLEN, CA, USA) was used for check of RNA purity; Qubit® RNA Assay Kit was used for measurement of RNA concentration; and the RNA Nano 6000 Assay Kit of the Agilent Bioanalyzer 2100 system (Agilent Technologies, CA, USA) was used for assessing of RNA integrity.

### Library preparation for transcriptome sequencing

A total amount of 3 μg RNA per sample was used as input material for the RNA sample preparations. Sequencing libraries were generated using NEBNext®Ultra™ RNA Library Prep Kit for Illumina® (NEB, USA) according to manufacturer’s recommendations. Briefly, mRNA was purified from total RNA by magnetic beads and cut randomly into short fragments by fragmentation buffer. First strand cDNA was synthesized by random hexamer primer and M-MuLV Reverse Transcriptase (RNase H-), using mRNA as a template. Second strand cDNA synthesis was subsequently performed using DNA Polymerase I and RNase H. The cDNA fragments of 150~ 200 bp in length were selected by AMPure XP beads, after end-repair and single nucleotide A (adenine) addition. Then, the cDNA library was obtained by PCR enrichment. At last, PCR products were purified and library quality was assessed on the Agilent Bioanalyzer 2100 system. The library preparations were sequenced on an Illumina Hiseq 4000 platform and paired-end reads were generated.

### Transcriptome assembly and gene functional annotation

The clean data were obtained by removing reads containing adapter and low quality reads from raw data. Transcriptome assembly was performed using Trinity [10] based on clean data with high quality. The sequences of unigenes were compared with Nr (NCBI non-redundant), Pfam (Protein family), Swiss-Prot (a manually annotated and reviewed protein sequence database), GO (Gene Ontology), COG (Cluster of orthologous groups), KOG (euKaryotic Orthologous Groups) and eggNOG (evolutionary genealogy of genes: Non-supervised Orthologous Groups) databases by BLAST, and the KEGG (Kyoto Encyclopedia of Genes and Genomes) Orthology results were obtained by comparing with KEGG using KOBAS2.0 [11]. After predicting the amino acid sequence of unigenes, we use HMMER [12] to compare them with Pfam database to get the annotation information of unigenes.

### Differential expression analysis

EdgeR program package was used for adjusting read counts of each sequenced library, and DEGseq (2010) R package was used for differential expression analysis of two samples. *P* value was adjusted using q value [13]. In this study, q value < 0.01 and |log2 (fold change)| > 1 were set as the threshold for significantly differential expression.

### Validation of RNA-Seq data by qRT-PCR

Expression of Ten DEGs in chrysanthemum was analyzed by qRT-PCR with three replicates to verify the RNA-Seq data. The qRT-PCR was performed using the SsoFast EvaGreen supermix (Bio-Rad, Hercules, CA, USA) and Bio-Rad CFX96™ detection system. A final 10 μL qRT-PCR reaction mixture contained 5 uL SsoFast EvaGreen supermix, 1 uL diluted cDNA sample, and 150 nM primers. Relative expression levels were calculated by the 2^−ΔΔCT^ method, and the Elongation Factor 1*α* (*EF1α*) gene (Forward primer: TTTTGGTATCTGGTCCTGGAG, Reverse primer: CCATTCAAGCGACAGACTCA) was used as a reference for quantitative expression analysis. The primers used in qRT-PCR are listed in Table 1.

**Table 1:** Primers of qRT-PCR for validation of the selected DEGs

Gene Name	Description	Primers
*MEKK1*	Mitogen-activated protein kinase kinase kinase	F:CAATTCGCGGAACACCAATG
		R:TTGAAACCGGGTCACTAACG
*MYB-like*	Myb-like DNA-binding domain	F:TGACCCTGATCCTGTGTTTG
		R:GTCTACCTCTCCCAATTCATCTG
*NAC90*	NAC domain-containing protein	F:TCCCACGACAAGAGAAACAAG
		R:TGATCCCAATGGCTCGATTAC
*FAD7*	omega-3 fatty acid desaturase	F:CTCCATTCCTTTCCACGGTAC
		R:GTTATGGGTCCGCTTCAAATG
*Hsp70*	Heat shock 70 kDa protein	F:CCAGCTCCACCTTGATACATC
		R:GAAGATTGAGGATGCGATTGATG
*LEA5*	Late embryogenesis abundant protein	F:AGAAAGTGTATCCGGAAGCG
		R:CGTCAACTTGGCTTGTTTGG
*LEA3*	Late embryogenesis abundant protein	F:GGGAAAGATAAGACAGGTGGG
		R:GGAGTACCCATACCCAAAGC
*P5CS*	pyrroline-5-carboxylate synthetase	F:TTAGCAGGTCTTTGTGGGTG
		R:GATGGGTGTTGAGAGGTAAAGG
*ABF*	ABA responsive element binding factor	F:CACCAAAGACTCCAATTCAACAG
		R:CAGAGGGAAGAGGAAAGTGTTG
*PP2C*	Protein phosphatase 2C	F:TTGTCGCAGTTCTCATACTCC
		R:TGATACGGCTTGTGGACTTG

### Determination of physiological indexes of chrysanthemum under low temperatures

Leaves of seedlings were used for determinations. Accumulation of H_2_O_2_ and O_2_^−^ was detected by histochemical staining, referring to Wang et al. (2017) [14]. Relative electrolyte conductivity (EC) was also measured following Wang et al. (2017) [14]. Peroxidase (POD) and catalase (CAT) activities were respectively measured following Ranieri et al. (2000) [15] and Zhang et al. (2011) [16]. Proline and soluble sugar contents were determinated following Irigoyen et al. (1992) [17] and Wang et al. (2013) [18]. Each determinations included three biological and technical replicates.

## Results

### Transcriptome sequencing and assembly

To comprehensively investigate the transcriptome and gene expression profiles of *D. grandiflorum* under normal and low temperature stress, four cDNA sample from leaves of chrysanthemum seedlings were prepared and sequenced using Illumina HiSeq 4000 platform. An overview of the RNA-Seq reads derived from the four libraries was presented in Table 2. After the low-quality reads were removed, 25.69Gb clean reads were obtained with an average of 6.42Gb (21.5 million) reads for each sample, and the percentage of Q30 base in each sample was not less than 96.33% (Additional file 1: Figure S1).

**Table 2:** Overview of the sequencing and assembly

Sample ID	Clean reads	Clean bases	Q30	GC content	Mapped reads	Unique match
T01	22,802,541	6,774,398,078	96.33%	43.62%	73.40%	51.43%
T02	21,839,313	6,501,753,240	96.52%	42.93%	73.77%	49.89%
T03	20,478,749	6,077,198,988	96.78%	42.90%	73.10%	47.49%
T04	21,323,634	6,339,923,198	96.68%	42.88%	71.71%	49.06%

With the Trinity program [10], a total of 199,754 transcripts were obtained from the clean reads with an N50 length of 1321 bp and a mean length of 878.8 bp. Among them, 93,837 unigenes were generated with an average length of 719.19 bp. The length distributions of transcripts and unigenes are given in Table 3. The clean data of each sample was compared with the transcript or unigene library. The proportion of mapped reads per library ranged from 71.71% to 73.77%, and unique mapped reads ranged from 47.49% to 51.43% (Table 2).

**Table 3:** Length distribution of the transcripts and unigenes clustered from de novo assembly

Length range	Transcript	Unigene
200–300	40,988 (20.52%)	30,475
300–500	44,661 (22.36%)	23,753
500–1000	54,466 (27.27%)	19,857
1000–2000	41,917 (20.98%)	13,504
2000^+^	17,722 (8.87%)	6248
Total number	199,754	93,837
Total length	175,543,436	67,486,480
N50 length	1321	1155
Mean length	878.8	719.19

### Gene annotation and functional classification

**Fig. 1:**
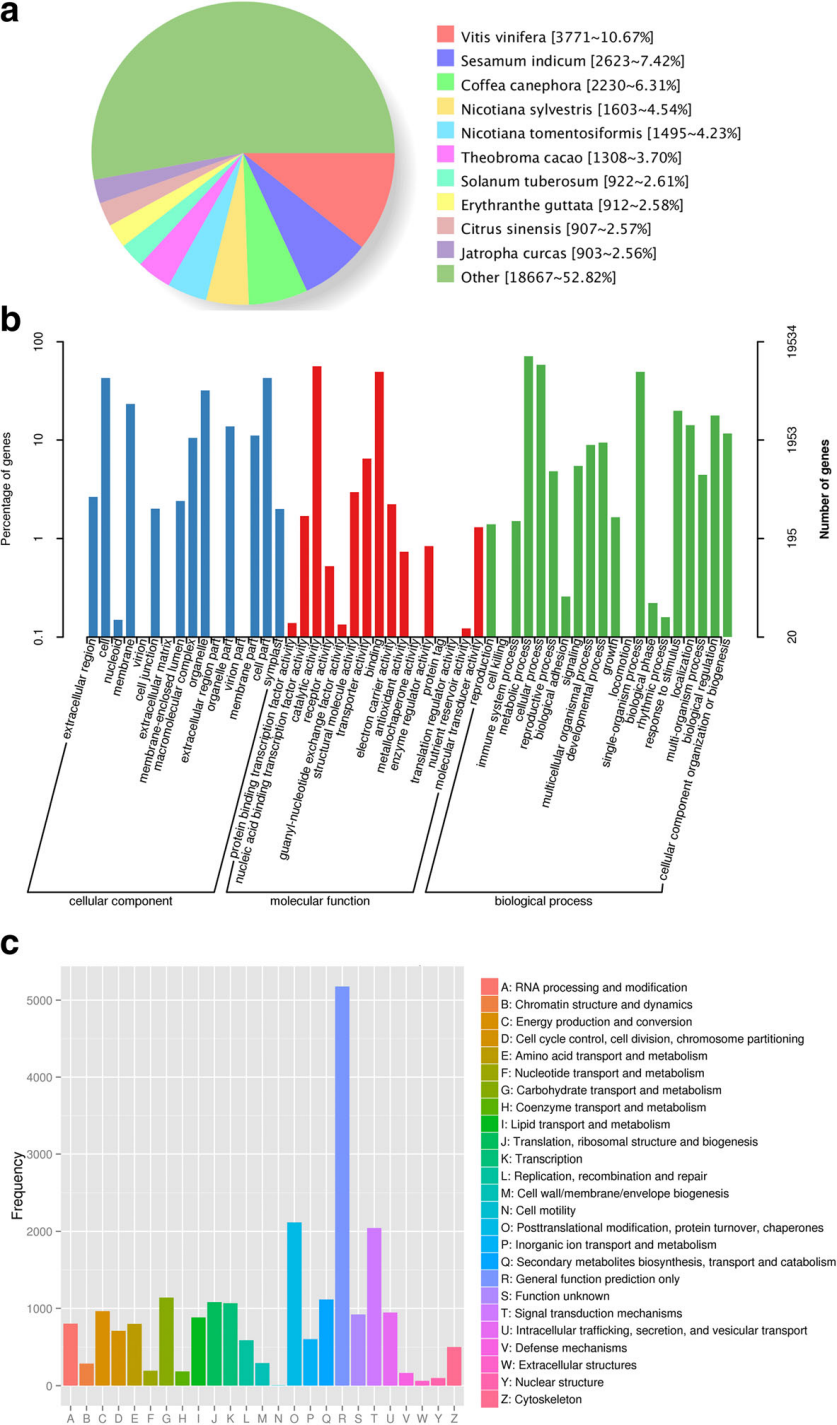
Functional annotation of assembled transcriptome. **a** Similarity of *D. grandiflorum* var. Jinba sequences with those of other species. **b** GO classification of the annotated unigenes. **c** KOG function classification of consensus sequence

To predict and analyze the function of the unigenes, we carried out functional annotation by using BLAST against multiple databases such as Nr, Swiss-Prot, GO, COG, KOG, KEGG, eggNOG, and Pfam. Of the 93,837 unigenes, 36,462 (38.86%) unigenes were successfully matched to homologous sequences in at least one of databases mentioned above. Among them, 10,226 (10.90%), 19,534 (20.82%), 12,584 (13.41%), 20,400 (21.74%), 24,137 (25.72%), 23,437 (24.98%), 33,201 (35.38%) and 35,370 (37.69%) unigenes were found in COG, GO, KEGG, KOG, Pfam, Swiss-Prot, eggNOG, and Nr databases, respectively. The Nr database produced the largest number of annotations. Compared with other species, chrysanthemum var. jinba showed the most matches to *Vitis vinifera* (3771), followed by *Sesamum indicum* (2623) and *Coffea canephora* (2230) (Fig. 1a).

GO assignments system was used to classify the possible functions of chrysanthemum genes. A total of 19,534 unigenes were successfully annotated and classified into 52 functional groups of three major GO categories (Cell Component (CC), Molecular Function (MF), and Biological Processes (BP)) (Fig. 1b). The top three GO terms for classified genes were ‘cell’ (8319), ‘cell part’(8319) and ‘organelle’ (6248) for cell component category; ‘catalytic activity’ (11,009), ‘binding’ (9622) and ‘transporter activity’ (1273) for molecular function; and ‘metabolic process’ (13,917), ‘cellular process’ (11,378) and ‘single-organism process’ (9623) for biological processes. In addition, genes involved in ‘membrane’, ‘response to stimulus’ and ‘biological regulation’ were also highly represent.

In total, 20,400 unigenes were grouped into 25 functional classifications based on KOG database (Fig. 1c). Among these classification, the largest group was ‘general function prediction only’ (5176), followed by ‘posttranslational modification, protein turnover, chaperones’ (2117), ‘signal transduction mechanisms’ (2046), ‘carbohydrate transport and metabolism’ (1140), ‘secondary metabolites biosynthesis, transport and catabolism’ (1115), ‘translation, ribosomal structure and biogenesis’ (1081), and ‘transcription’ (1067).

### Analysis of potential differentially expressed genes (DEGs)

The clean reads from each sample were compared to the unigene library, and gene expression levels were estimated using RSEM (RNA-Seq by Expectation Maximization) according to the results of comparison. FPKM (fragments per kilobase of transcript per million fragments mapped) values were used to indicate the expression abundance of unigenes.

**Fig. 2:**
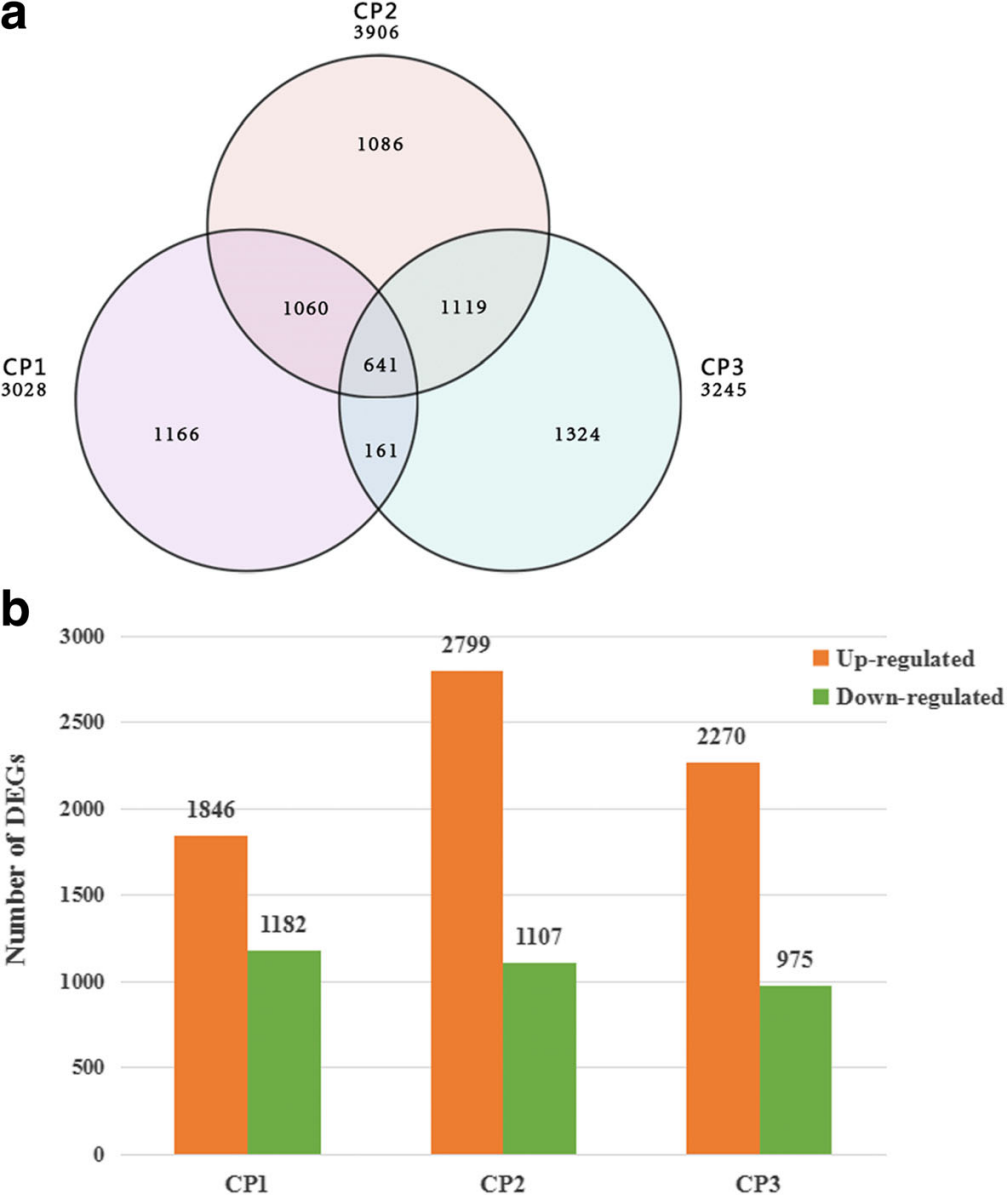
Venn diagram and histogram of DEGs during low temperature stresses. **a** Venn diagram showing DEGs expressed at each of the three low temperature treatments. **b** The numbers of DEGs identified in comparisons between pairs of libraries

From three comparison, including CP1 (T01 vs T02), CP2 (T01 vs T03) and CP3 (T01 vs T04), a large number of DEGs were identified. The number of DEGs detected was as follows: CP1 3028 (1846 up- and 1182 down-regulated), CP2 3906 (2799 and 1107) and CP3 3245 (2270 and 975) (Fig. 2b). We found that more DEGs up regulated in treatment T03 which underwent CA than the treatment T04 without CA. Moreover, the venn diagram was used for analyses of unique and overlapping sets of DEGs in each treatment. As shown in Fig. 2a, there were 641 genes differentially expressed in all comparisons, indicating that these genes involved in the process of plants response to both chilling and freezing stresses, which were of great value for low temperature tolerance study. We also identified 1166, 1086 and 1324 DEGs expressed only in T02, T03 and T04, respectively. Thus it can be seen that there were 1166 genes only playing roles in the process of CA, not working in freezing period, and 1324 genes involved in response to rapid freezing. Of total 6557 DEGs, 4378 genes were successfully annotated with eight databases. The number of annotated DEGs in each treatment is shown in Table 4.

**Table 4:** DEGs annotation of *D. grandiflorum* var. Jinba

	CP1	CP2	CP3
Total annotated	2280	2530	2166
COG	813	796	562
GO	1335	1390	1101
KEGG	827	832	596
KOG	1311	1332	990
Pfam	1825	1966	1627
Swiss-Prot	1654	1813	1534
eggNOG	2181	2400	2040
Nr	2257	2497	2140

### KEGG pathway analysis for DEGs

KEGG annotation was used for DEGs from the above comparisons. In the CP1, 493 DEGs were assigned to the KEGG database involved 108 pathways; for CP2, 519 DEGs were assigned to 113 pathways; and for CP3, 386 DEGs assigned to 105 pathways. The most reliable top ten significantly enriched pathways of DEGs under low temperature treatments were represented as Table 5.

**Table 5:** The most reliable top ten significantly enriched pathways of DEGs under low temperature treatments

	Pathway ID	Pathway	DEGs in pathway	All genes in pathway	*P*-value
CP1	ko03008	Ribosome biogenesis in eukaryotes	47	137	5.59E-13
	ko00196	Photosynthesis - antenna proteins	20	64	3.19E-09
	ko00940	Phenylpropanoid biosynthesis	38	233	2.53E-07
	ko00500	Starch and sucrose metabolism	42	301	4.29E-06
	ko00052	Galactose metabolism	17	104	0.0005392
	ko00400	Phenylalanine, tyrosine and tryptophan biosynthesis	13	78	0.0019789
	ko00906	Carotenoid biosynthesis	8	38	0.0032878
	ko00130	Ubiquinone and other terpenoid-quinone biosynthesis	10	66	0.0123906
	ko00603	Glycosphingolipid biosynthesis - globo series	3	8	0.0132652
	ko00360	Phenylalanine metabolism	17	143	0.0156871
CP2	ko03008	Ribosome biogenesis in eukaryotes	31	137	4.55E-09
	ko00196	Photosynthesis - antenna proteins	20	64	7.76E-09
	ko04626	Plant-pathogen interaction	45	272	5.72E-08
	ko00940	Phenylpropanoid biosynthesis	29	233	0.0020767
	ko00564	Glycerophospholipid metabolism	20	149	0.0042047
	ko00360	Phenylalanine metabolism	19	143	0.0058143
	ko00052	Galactose metabolism	15	104	0.0064176
	ko00500	Starch and sucrose metabolism	33	301	0.0079662
	ko04075	Plant hormone signal transduction	38	360	0.0084879
	ko00740	Riboflavin metabolism	3	8	0.0152681
CP3	ko00196	Photosynthesis - antenna proteins	28	64	0
	ko04626	Plant-pathogen interaction	33	272	5.61E-06
	ko04075	Plant hormone signal transduction	39	360	1.24E-05
	ko00860	Porphyrin and chlorophyll metabolism	14	77	4.17E-05
	ko04712	Circadian rhythm - plant	11	55	0.0001138
	ko00906	Carotenoid biosynthesis	8	38	0.0006843
	ko00195	Photosynthesis	15	114	0.0009170
	ko00740	Riboflavin metabolism	3	8	0.0067278
	ko00591	Linoleic acid metabolism	6	36	0.0106150
	ko00564	Glycerophospholipid metabolism	14	149	0.0256548

Integrate these three comparisons, the major pathways involved in the cold response mechanism were ‘Starch and sucrose metabolism’, ‘Galactose metabolism’, ‘Glycerophospholipid metabolism’, ‘Biosynthesis of unsaturated fatty acids’, ‘Plant hormone signal transduction’ and ‘Plant-pathogen interaction’ (Additional file 1: Figure S2). As shown in Table 6, for first four biological metabolism and synthesis pathways, the number of DEGs in CP1 was more than CP2 or CP3; but for last two signal transduction related pathways, DEGs number of CP1 was less than CP2 or CP3.

**Table 6:** DEG statistics in five major pathways

#Pathways	CP1	CP2	CP3
Starch and sucrose metabolism	42	33	22
Galactose metabolism	17	15	6
Glycerophospholipid metabolism	14	20	14
Biosynthesis of unsaturated fatty acids	7	9	1
Plant hormone signal transduction	29	38	39
Plant-pathogen interaction	19	45	33

### Protein kinase (PK) and transcription factors (TFs) responding to low temperature

**Fig. 3:**
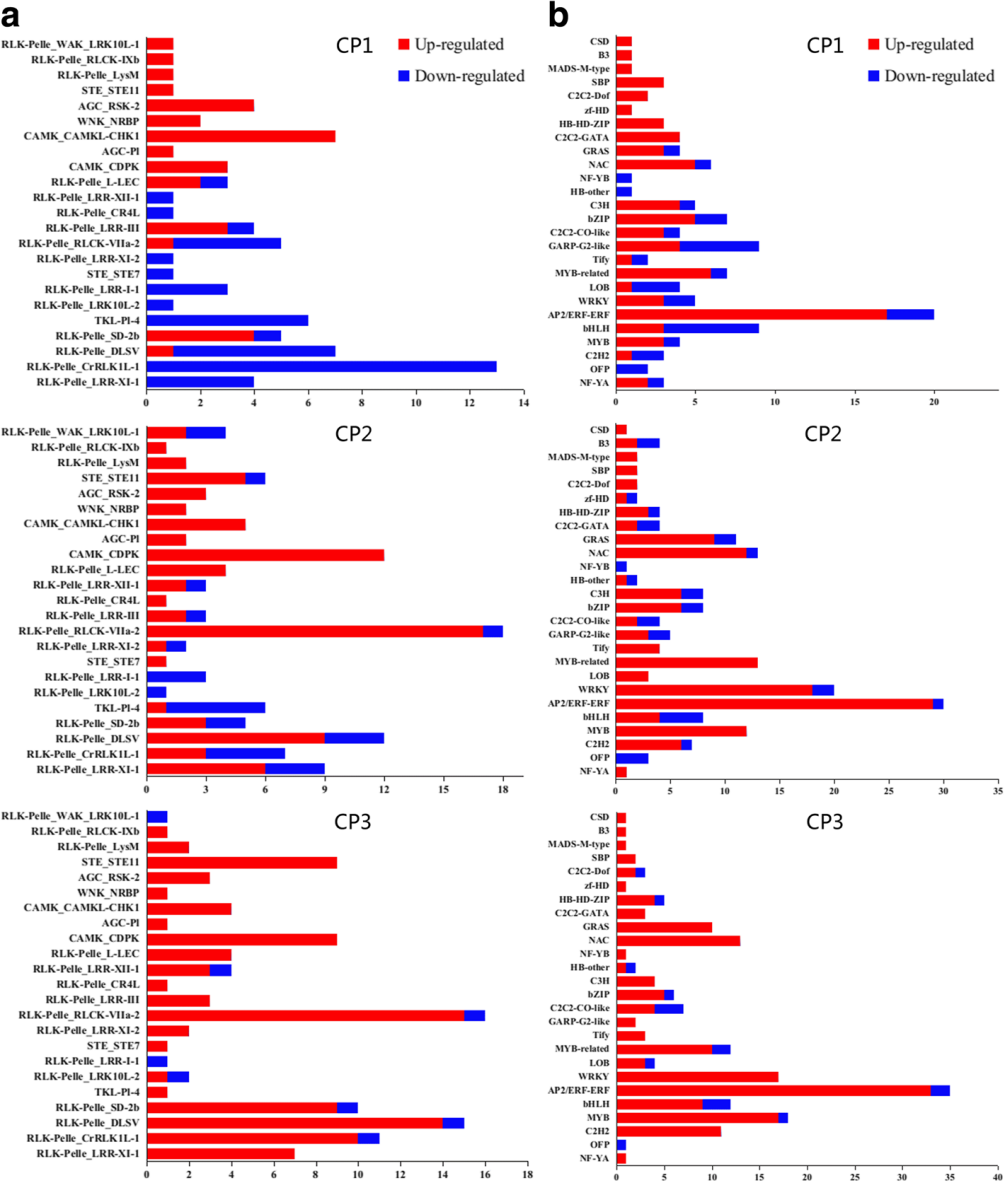
Differentially expressed protein kinases **a** and transcription factors **b** responsive to low temperature. Within each bar, number of up- and down-regulated genes is shown in red and blue, respectively

A total of 96, 131, and 129 DEGs encoding protein kinases belonging to 23 major families were found in CP1, CP2 and CP3 treatments, respectively. Among them, 51 (14 up- and 37 down-regulated), 71 (50 and 21) and 80 (71 and 9) receptor-like kinases (RLKs) were identified. And there were 4 (2 up- and 2 down-regulated), 8 (7 and 1) and 10 (10 and 0) genes of CP1, CP2 and CP3 involved in the mitogen-activated protein kinase (MAPK) cascade. We found 3, 12 and 9 DEGs of calcium-dependent protein kinase (CDPK) family and 7, 5 and 4 DEGs of CBL-interacting protein kinase (CIPK) family were all up regulated in CP1, CP2 and CP3. Moreover, we identified 13, 12 and 11 protein phosphatase (PP) differentially expressed in three treatments. The details of these PK and PP genes are shown in Additional file 2: Table S1. These results indicated that protein phosphorylation may be induced by low temperature in chrysanthemum. In addition, it was interesting that there were more up-regulated PK in CP2 and CP3 than CP1 (Fig. 3a).

In this study, totally 123 (84 up- and 39 down-regulated), 190 (157 and 33) and 193 (172 and 21) transcription factor (TF) genes were identified in CP1, CP2 and CP3. Obviously, the number of up- regulated expressed transcription TFs in CP2 and CP3 was greater than that of CP1 (Fig. 3b). A large number of TF families were involved in low temperature response. Generally, the major TF families were AP2/ERF, WRKY, bHLH, MYB, MYB-related, and NAC. In treatment CP1, the AP2/ERF family was the largest group (18%), followed by the bHLH family (8%) and the GARP-G2-like family (6%); for CP2, the top three family were AP2/ERF (17%), WRKY (12%) and MYB (7%); and for CP3, the top three were AP2/ERF (20%), WRKY (10%), MYB (10%) (Additional file 1: Figure S3). Of AP2/ERF genes, 17, 29 and 33 were up-regulated in CP1, CP2 and CP3, respectively. And the members of WRKY and MYB families were more active in freezing condition rather than in chilling condition.

### Gene related to the membrane system and osmoregulation

The increase of membrane lipids unsaturation level can increase the fluidity of membrane, accordingly improving the cold resistance of plants [19]. In our study, we identified 12 lipid-transfer proteins (LTPs) and 7 fatty acid desaturase (FADs) genes. Among them, 5 LTPs and 3 FADs were up-regulated in CP1, 7 LTPs and 5 FADs up-regulalted in CP2, and only 4 LTPs and 1 FADs were up-regulated in CP3. The details of genes related to the biofilm system are shown in Table 7.

**Table 7:** Gene related to the biofilm system in response to low temperature

	Gene ID	CP1 log2FC	CP1 regulated	CP2 log2FC	CP2 regulated	CP3 log2FC	CP3 regulated	Gene description
LTPs	c98772.graph_c0	2.06	up	2.26	up	0.38	normal	Non-specific lipid transfer protein
	c94507.graph_c0	0.43	normal	−0.12	normal	1.13	up	Non-specific lipid transfer protein
	c90293.graph_c0	4.24	up	4.30	up	1.47	normal	Non-specific lipid-transfer protein-like protein
	c86130.graph_c0	4.39	up	4.66	up	1.53	normal	Lipid transfer-like protein
	c96305.graph_c0	1.50	normal	2.41	up	1.28	normal	Non-specific lipid-transfer protein-like protein
	c93927.graph_c1	3.17	normal	3.48	up	–	–	Probable non-specific lipid-transfer protein-like protein
	c111292.graph_c1	3.44	up	3.97	up	1.36	up	Non-specific lipid-transfer protein-like protein
	c95472.graph_c0	0.93	normal	1.38	normal	2.16	up	Non-specific lipid-transfer protein
	c45235.graph_c0	3.55	up	2.77	normal	3.83	up	Putative lipid-transfer protein
	c91182.graph_c0	0.78	normal	0.86	normal	−1.50	down	Lipid transfer protein EARLI 1
	c92863.graph_c0	2.98	normal	3.81	up	–	–	Non-specific lipid-transfer protein-like protein
	c95124.graph_c0	−0.95	normal	−1.41	normal	−3.08	down	Lipid transfer protein EARLI 1
FADs	c103089.graph_c1	3.38	up	2.95	up	−0.09	normal	Omega-3 fatty acid desaturase
	c98666.graph_c0	3.35	up	2.63	up	0.11	normal	Omega-3 fatty acid desaturase
	c86611.graph_c0	1.84	up	1.59	normal	0.21	normal	Omega-3 fatty acid desaturase
	c87665.graph_c0	1.21	normal	2.50	up	0.56	normal	Omega-6 fatty acid desaturase
	c87665.graph_c1	1.02	normal	2.44	up	0.88	normal	Omega-6 fatty acid desaturase
	c86525.graph_c0	0.64	normal	2.02	up	−0.58	normal	Omega-6 fatty acid desaturase
	c106808.graph_c0	−0.07	normal	0.82	normal	1.26	up	Omega-6 fatty acid desaturase
PLDs	c105153.graph_c0	0.03	normal	0.95	normal	1.10	up	Phospholipase D alpha 1
	c112648.graph_c0	3.42	up	3.67	up	1.43	up	Phospholipase D alpha 1
	c115302.graph_c0	3.50	up	4.51	up	0.49	normal	Phospholipase D p1
	c98821.graph_c0	2.89	up	3.39	up	0.97	normal	Phospholipase D alpha 1
	c114556.graph_c0	−2.12	down	−3.02	down	−0.19	normal	phospholipase D Z-like
	c113287.graph_c0	−0.20	normal	2.22	up	0.71	normal	Phospholipase D beta 1
SPLA2	c116204.graph_c1	−0.83	normal	1.78	normal	1.59	up	Triacylglycerol lipase SDP1
	c96544.graph_c0	1.75	up	0.35	normal	2.09	up	Phospholipase A2-alpha
	c105515.graph_c0	2.17	up	1.65	normal	0.94	normal	Patatin-like phospholipase
POD	c96977.graph_c0	–	–	3.80	up	2.84	up	Peroxidase N1
	c103682.graph_c0	4.29	up	3.85	up	4.27	up	Peroxidase 17
	c106645.graph_c0	3.36	up	3.94	up	1.70	normal	Cationic peroxidase 1
	c113544.graph_c0	−2.75	down	−0.11	normal	−0.22	normal	Peroxidase N1
	c100631.graph_c0	–	–	3.97	up	4.86	up	Lignin-forming anionic peroxidase
	c114296.graph_c0	−0.51	normal	−1.66	normal	−2.82	down	Peroxidase 64
	c90653.graph_c0	−3.06	down	−0.21	normal	0.24	normal	Peroxidase (Precursor)
	c98404.graph_c0	0.17	normal	0.26	normal	−2.07	down	Lignin-forming anionic peroxidase (Precursor)
	c99288.graph_c0	−2.62	down	−2.34	down	−0.69	normal	Peroxidase 12

According to the pathway enrichment analysis, ‘starch and sucrose metabolism’ (ko00500), ‘galactose metabolism’ (ko00052), ‘arginine and proline metabolism’ (ko00330) involved plenty of genes that regulating and relieving the osmotic stress caused by cold-evoked dehydration. In the treatment CP1, 7 starch-degrading genes, 4 cellulose-degrading genes, 3 sucrose-phosphate synthase (SPS) genes, 2 trehalose phosphate synthase (TPS) genes, 3 trehalose phosphatase (TPP) genes, 3 galactinol synthase genes, 4 raffinose synthase gene and 1 pyrroline-5-carboxylate synthetase (P5CS) gene were up-regulated; an equivalent order for CP2 were 7, 3, 3, 2, 3, 3, 4 and 1; for CP3 were 4, 1, 0, 0, 2, 1, 3 and 0. Evidently, under freezing conditions, activities of starch and cellulose degradation and above soluble sugars syntheses were decreased in chrysanthemum without cold acclimation (T04). The details of genes related to osmotic regulation system are shown in Table 8.

**Table 8:** Gene related to osmotic regulation system in response to low temperature

	Gene ID	CP1 log2FC	CP1 regulated	CP2 log2FC	CP2 regulated	CP3 log2FC	CP3 regulated	Gene description
Starch-degrading genes	c106047.graph_c0	1.82	normal	2.10	up	0.57	normal	Beta-amylase
	c111660.graph_c0	4.57	up	4.51	up	4.83	up	Beta-amylase
	c98302.graph_c0	3.25	up	3.61	up	1.87	up	Beta-amylase
	c111420.graph_c0	2.83	up	3.22	up	1.81	up	Beta-amylase
	c111420.graph_c1	2.83	up	3.22	up	1.58	up	Beta-amylase
	c102389.graph_c0	2.43	up	1.99	up	0.97	normal	Probable alpha-amylase
	c116071.graph_c0	2.01	up	1.94	normal	−0.90	normal	Alpha-amylase
	c116145.graph_c0	1.93	up	2.06	up	0.02	normal	Alpha-amylase
Cellulose-degrading genes	c111098.graph_c0	2.59	up	2.32	up	1.37	normal	Beta glucosidase 41 isoform 3
	c91317.graph_c0	2.74	up	2.02	normal	0.98	normal	Raucaffricine-O-beta-D- glucosidase
	c108565.graph_c1	1.88	up	1.43	normal	0.26	normal	lysosomal beta glucosidase-like
	c96136.graph_c0	2.96	up	2.90	up	0.33	normal	Beta-glucosidase 18
	c106631.graph_c0	−2.07	down	−1.52	normal	0.23	normal	Strictosidine-O-beta-D- glucosidase
	c104722.graph_c1	−2.20	down	−2.06	normal	−0.81	normal	Probable beta-D-xylosidase 5
	c93973.graph_c0	−3.35	down	−1.34	normal	−0.06	normal	lysosomal beta glucosidase-like isoform X1
	c98430.graph_c0	−1.82	down	−1.98	normal	−0.44	normal	Beta-glucosidase 18
	c98430.graph_c1	−2.07	down	−2.41	down	−0.82	normal	beta-glucosidase 18 isoform X1
	c104480.graph_c0	0.12	normal	2.23	up	0.56	normal	Probable beta-D-xylosidase 5
	c112478.graph_c0	1.49	normal	1.12	normal	1.12	up	Beta-glucosidase 11
	c114139.graph_c0	−1.23	normal	−1.96	normal	−1.23	down	Beta-glucosidase 18
SPS	c114821.graph_c1	3.09	up	3.44	up	0.34	normal	sucrose phosphate synthase
	c114715.graph_c0	2.56	up	2.48	up	−0.88	normal	sucrose phosphate synthase
	c114821.graph_c0	3.67	up	4.00	up	0.92	normal	sucrose phosphate synthase
SS	c103323.graph_c0	−1.84	normal	−1.53	normal	1.11	up	Sucrose synthase 3
TPS	c102854.graph_c0	−4.99	down	−2.01	normal	−1.08	normal	Probable alpha,alpha-trehalose- phosphate synthase
	c104136.graph_c0	−4.61	down	−1.25	normal	−0.91	normal	Probable alpha,alpha-trehalose- phosphate synthase
	c115624.graph_c0	−2.17	down	−0.23	normal	−0.63	normal	trehalose-6-phosphate synthase
	c92114.graph_c0	2.38	normal	3.25	up	–	–	trehalose-7-phosphate synthase
	c112854.graph_c0	−3.03	down	−1.39	normal	−1.34	down	Probable alpha,alpha-trehalose- phosphate synthase
	c113270.graph_c0	−2.41	down	1.08	normal	0.44	normal	Probable alpha,alpha-trehalose- phosphate synthase
	c93906.graph_c0	−4.25	down	−2.27	normal	−0.82	normal	Probable alpha,alpha-trehalose- phosphate synthase
	c99330.graph_c0	3.86	up	3.89	up	–	–	Alpha,alpha-trehalose- phosphate synthase
	c107046.graph_c1	−1.62	normal	−2.72	down	−0.88	normal	trehalose 6-phosphate synthase
	c108202.graph_c0	−3.46	down	0.56	normal	−0.17	normal	Probable alpha,alpha-trehalose- phosphate synthase
	c94647.graph_c0	2.41	up	1.49	normal	–	–	Alpha,alpha-trehalose- phosphate synthase
	c86304.graph_c0	−3.60	down	−1.52	normal	−0.57	normal	Probable alpha,alpha-trehalose- phosphate synthase
TPP	c110653.graph_c0	3.26	up	5.87	up	5.00	up	trehalose-phosphate phosphatase
	c116316.graph_c0	1.99	up	2.15	up	1.00	normal	trehalose-phosphate phosphatase
	c104330.graph_c0	3.07	up	3.06	up	2.73	up	trehalose-phosphate phosphatase
Raffinose synthase genes	c80434.graph_c0	2.58	up	3.34	up	1.41	normal	Galactinol synthase
	c98797.graph_c0	4.00	up	5.46	up	1.90	up	Galactinol synthase
	c81587.graph_c0	2.44	up	2.55	up	0.97	normal	Galactinol synthase
	c115834.graph_c0	4.05	up	7.49	up	4.99	up	Probable galactinol--sucrose galactosyltransferase
	c115556.graph_c0	−4.49	down	1.01	normal	1.97	up	Probable galactinol--sucrose galactosyltransferase
galM	c98143.graph_c0	−1.78	down	−1.26	normal	−0.44	normal	Aldose 1-epimerase
galA	c110075.graph_c0	−2.00	down	−2.54	down	−0.43	normal	Alpha-galactosidase
P5CS	c110924.graph_c0	2.76	up	2.95	up	0.74	normal	pyrroline-5-carboxylate synthetase

Moreover, we found a group of cold-related proteins, including cold-regulated (COR), cold shock and heat shock proteins (HSPs), which were invovled in the low temperature stress response process. Among them, HSPs accounted for a large proportion. And we also identified some anti-freezing proteins (AFPs) [7], such as beta-1,3-glucanase proteins (GLPs), thaumatin-like proteins (TLPs), polygalacturonase-inhibiting proteins (PGIPs) and late-embryogenesis-abundant proteins (LEAs). In total, there were 14, 17 and 13 AFPs-related genes up-regulated in CP1, CP2 and CP3, respectively. These genes are shown as Table 9.

**Table 9:** Genes encoding cold-related proteins and anti-freezing proteins

	Gene ID	CP1 log2FC	CP1 regulated	CP2 log2FC	CP2 regulated	CP3 log2FC	CP3 regulated	Gene description
COR	c98333.graph_c1	–	–	4.07	up	4.47	up	putative cold-inducible protein
	c97739.graph_c0	3.41	up	4.12	up	1.01	normal	cold-responsive protein
	c99607.graph_c0	3.15	up	1.50	normal	3.31	up	Cold regulated gene 27
Cold shock proteins	c113528.graph_c0	2.83	up	2.73	up	0.01	normal	Cold shock protein 1
	c94983.graph_c0	2.47	up	2.52	up	–	–	Cold shock protein 1
	c99900.graph_c0	1.78	up	1.19	normal	0.37	normal	Cold shock domain-containing protein 3
AFPs	c99499.graph_c0	−1.26	normal	−2.87	down	−0.40	normal	beta-1,3-glucanase
	c106137.graph_c0	0.85	normal	2.24	up	1.83	up	beta-1,3-glucanase
	c101035.graph_c0	−2.55	down	−3.21	down	0.55	normal	chitinase 2-like
	c82667.graph_c0	4.46	up	8.71	up	5.59	up	Thaumatin-like protein
	c82207.graph_c0	−3.00	down	−1.66	normal	− 1.40	normal	Thaumatin-like protein
	c107884.graph_c0	3.81	up	4.24	up	2.17	up	Thaumatin-like protein 1
	c100473.graph_c1	−2.06	down	−1.79	normal	−0.31	normal	Thaumatin-like protein 1a
	c97008.graph_c0	−3.44	down	−2.88	normal	−1.77	normal	Thaumatin-like protein 1
	c96509.graph_c0	5.01	up	4.94	up	0.41	normal	polygalacturonase-inhibiting protein 4
	c92070.graph_c0	7.56	up	8.54	up	–	–	Late embryogenesis abundant protein
	c70366.graph_c0	5.37	up	5.74	up	0.05	normal	Late embryogenesis abundant protein
	c58403.graph_c0	4.52	up	5.52	up	0.24	normal	Late embryogenesis abundant protein
	c105364.graph_c0	1.15	normal	7.32	up	6.28	up	Late embryogenesis abundant protein
	c114597.graph_c0	0.27	normal	1.97	up	2.85	up	Late embryogenesis abundant protein
	c105750.graph_c0	2.76	up	2.12	normal	–	–	Late embryogenesis abundant protein
	c100124.graph_c0	2.77	up	2.27	up	−0.52	normal	Late embryogenesis abundant protein
	c110317.graph_c0	1.76	up	3.51	up	2.82	up	Late embryogenesis abundant protein
	c94818.graph_c0	–	–	10.01	up	9.79	up	Late embryogenesis abundant protein
	c78821.graph_c0	3.23	up	7.04	up	6.42	up	Late embryogenesis abundant protein
	c102635.graph_c0	2.79	up	1.32	normal	−0.25	normal	Late embryogenesis abundant protein
	c108163.graph_c0	3.73	up	4.59	up	2.68	up	Late embryogenesis abundant protein
	c112429.graph_c0	1.99	up	3.51	up	2.45	up	Late embryogenesis abundant protein
	c96108.graph_c0	1.09	normal	6.29	up	5.22	up	Late embryogenesis abundant protein
	c114782.graph_c0	0.56	normal	1.61	normal	2.22	up	Late embryogenesis abundant protein
	c108048.graph_c2	6.00	up	6.95	up	4.39	up	Late embryogenesis abundant protein
HSPs	c108834.graph_c0	−0.67	normal	4.29	up	3.33	up	Heat shock protein
	c100907.graph_c0	0.01	normal	2.85	up	2.48	up	Heat shock protein
	c120647.graph_c0	–	–	–	–	3.32	up	Heat shock protein 70
	c104201.graph_c0	1.36	normal	2.04	up	−0.07	normal	Heat shock 70 kDa protein
	c113668.graph_c0	−1.70	normal	−2.63	down	−0.89	normal	Heat shock 70 kDa protein
	c97547.graph_c1	4.43	up	5.32	up	1.26	up	Heat shock 70 kDa protein
	c86961.graph_c0	−0.15	normal	2.23	up	1.37	up	Heat shock cognate 70 kDa protein
	c82373.graph_c0	–	–	3.05	up	–	–	Heat shock cognate 70 kDa protein
	c108184.graph_c0	0.93	normal	2.56	up	1.01	normal	Heat shock cognate 70 kDa protein
	c107360.graph_c0	0.74	normal	2.23	up	1.51	up	Heat shock factor protein HSF24
	c98633.graph_c0	0.03	normal	1.28	normal	1.10	up	Heat shock cognate 70 kDa protein
	c88835.graph_c0	−1.57	normal	2.03	up	1.90	up	Heat shock cognate 70 kDa protein
	c114383.graph_c0	0.88	normal	3.24	up	2.47	up	Heat shock cognate 70 kDa protein
	c114642.graph_c0	1.03	normal	2.34	up	−0.50	normal	Heat shock 70 kDa protein
	c106065.graph_c0	2.03	up	2.13	normal	0.51	normal	Heat shock cognate protein
	c112665.graph_c0	0.39	normal	3.17	up	1.91	up	Heat shock cognate 70 kDa protein
	c95742.graph_c0	3.89	up	3.90	up	−0.30	normal	Heat shock 70 kDa protein
	c111753.graph_c0	−0.02	normal	2.82	up	1.95	up	Heat shock cognate 70 kDa protein
	c81956.graph_c0	−1.60	normal	−2.41	down	−1.84	down	Stromal 70 kDa heat shock-related protein
	c97818.graph_c0	−2.43	down	−2.06	down	−0.93	normal	15.4 kDa class V heat shock protein
	c91820.graph_c0	−1.35	normal	−2.66	down	−1.93	down	Stromal 70 kDa heat shock-related protein
	c96991.graph_c0	−2.12	down	−2.42	down	−2.23	down	Stromal 70 kDa heat shock-related protein
	c113713.graph_c1	−0.33	normal	−1.35	normal	1.65	up	22.7 kDa class IV heat shock protein
	c102754.graph_c0	3.00	up	3.17	up	0.01	normal	Heat shock cognate protein 80
	c112282.graph_c0	4.18	up	4.97	up	1.27	up	Heat shock cognate 70 kDa protein
	c115107.graph_c2	3.11	up	3.26	up	0.16	normal	Heat shock protein 90–2
	c102959.graph_c0	3.21	up	3.31	up	−0.06	normal	Heat shock 70 kDa protein
	c112881.graph_c0	2.46	up	2.55	up	−0.20	normal	Stromal 70 kDa heat shock-related protein
	c58792.graph_c0	–	–	4.51	up	3.93	up	Small heat shock protein
	c115107.graph_c0	1.87	up	1.89	normal	0.12	normal	Heat shock protein 90–2
	c109478.graph_c0	0.06	normal	2.11	up	1.02	normal	Heat shock cognate 70 kDa protein
	c99010.graph_c0	−1.34	normal	−2.22	down	−0.64	normal	15.7 kDa heat shock protein

### Plant hormone signal transduction pathway

A total of 68 DEGs were invovled in several plant hormone signal transduction pathways, such as auxin, cytokinin (CK), gibberellin (GA), abscisic acid (ABA), ethylelne (ET), brassinosteroid (BR), jasmonic acid (JA) and salicylic acid (SA) in our study. Among them, auxin, ABA, ET and JA pathways were predominatly induced. In the auxin signaling pathway, there were 9, 10 and 9 genes up-regulated in T02, T03 and T04; in the CK pathway, the number of up-regulated genes in three treatments was 4, 3 and 2; in the ET pathway, the number was 2, 2 and 7; and in the JA pathway, the number was 1, 5 and 7. In ABA signaling pathway, totally 4 ABA receptor genes, 1 ABA-responsive element binding factor (ABF) gene, 4 serine/threonine-protein kinase SRK2 (SnRK2) genes, and 6 protein phosphatase 2C (PP2C) genes were differentially expressed during low temperature conditions. Unexpectedly, under the chilling condition, there was no gene differentially expressed in the SA signaling pathway, while one gene up-regulated under freezing conditions. The above results suggested that plant hormone played important roles in response to low temperature stress in chrysanthemum.

### Verification of DEGs using qRT-PCR

**Fig. 4:**
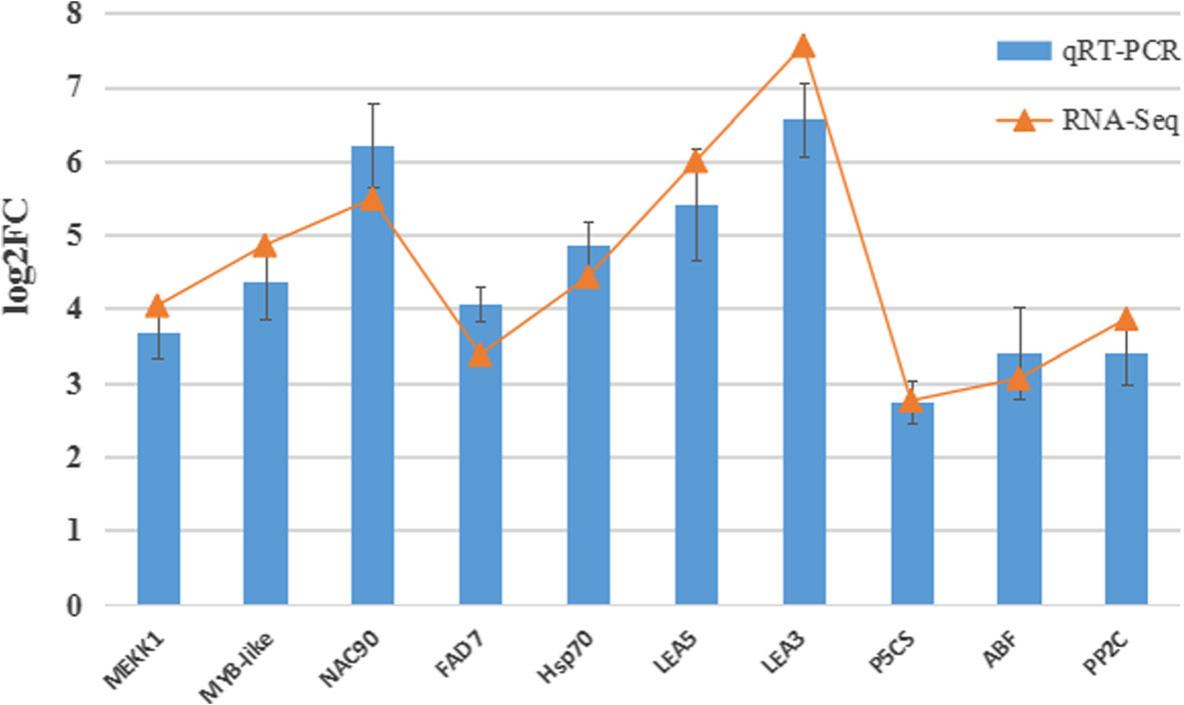
The relative expression levels of ten DEGs identified in the comparison CP1 between RNA-Seq and qRT-PCR. The genes relative expression levels were determined by 2^−ΔΔCT^ as expressed, and were normalized to the expression level of *EF1α*

To further verify the expression levels of genes obtained from Illumina RNA-Seq platform, we have selected ten genes induced by low temperature to perform qRT-PCR, including genes encoding protein kinases, transcription factors, fatty acid desaturase, heat shock protein, late-embryogenesis-abundant protein, pyrroline-5-carboxylate synthetase, ABA-responsive element binding factor and polygalacturonase-inhibiting protein. The results of experiment showed a strong correlation with the RNA-Seq data (Fig. 4).

### Analyses of phenotypic and physiological changes of chrysanthemum at different low temperatures.

**Fig. 5:**
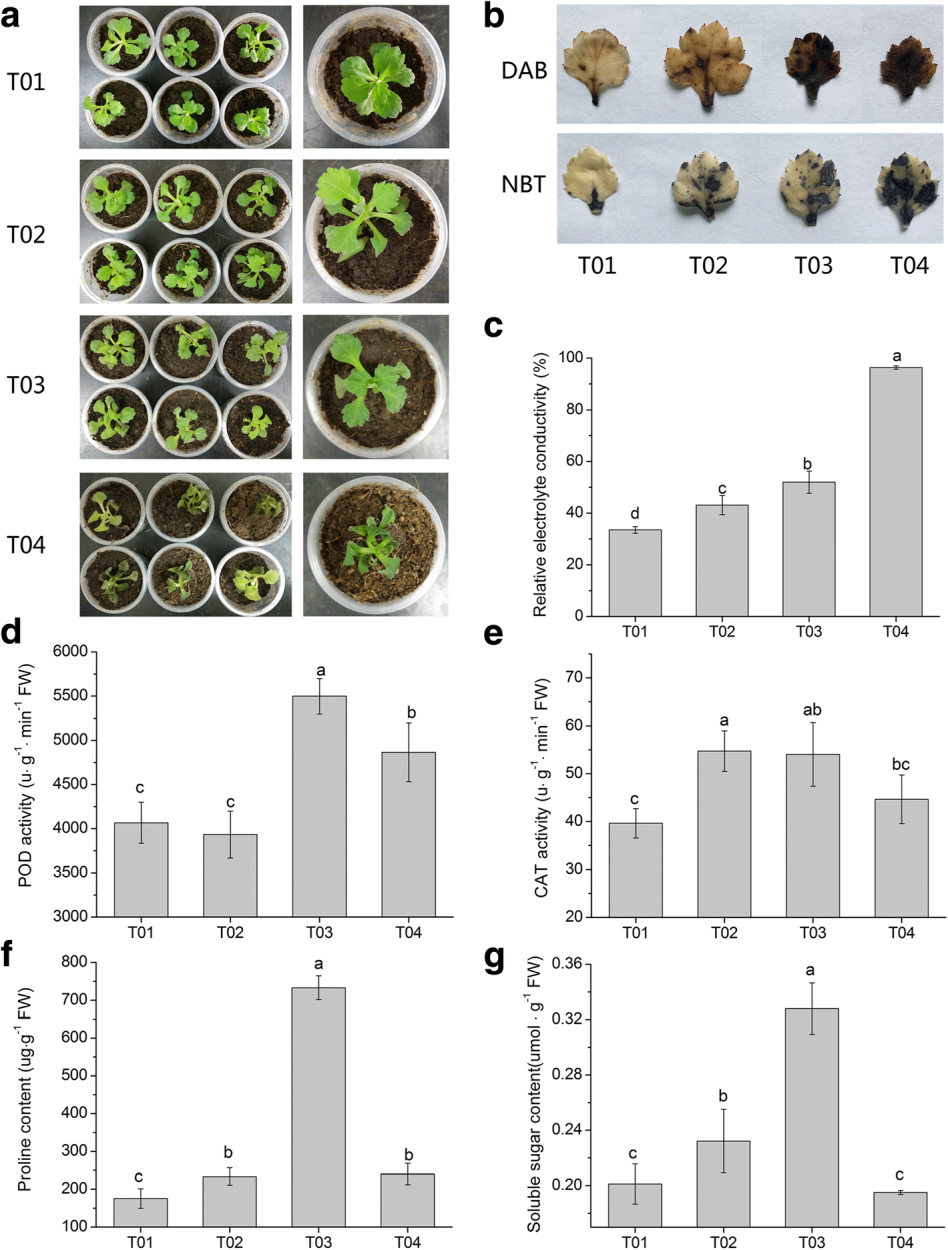
Phenotypic and physiological changes of chrysanthemum at different low temperatures. **a** Phenotypic comparison of chrysanthemum in four different treatments. **b** Histochemical staining with DAB and NBT for assessing the accumulation of H_2_O_2_ and O_2_^−^, respectively, under low temperatures. **c-g** Analysis of relative electrolyte conductivity **c**, POD activity **d**, CAT activity **e**, proline content **f**, and soluble sugar **g** under low temperatures. Data represent means and standard errors of three replicates. The different letters above the columns indicate significant (*P* < 0.05) differences according to Duncan’s multiple range test

As shown in Fig. 5a, the phenotypes of chrysanthemum seedlings under chilling stress (4 °C) was not significantly different from those under normal conditions, but only the youngest leaves of individual seedlings appeared dying. And under freezing stress (− 4 °C), it was clear that the seedlings without prior CA (T04) were more wilted than those with CA (T03). Under low temperature, the membrane permeability of chrysanthemum leaves increased and electrolyte leakage occurred. The degree of electrolyte extravasation was higher in chrysanthemum under freezing conditions than that under the chilling condition (Fig. 5c). And the electrolyte conductivity of T03 was lower than T04, suggesting that CA could increase the tolerance of chrysanthemum to freezing stress.

In order to further explore the oxidative damage of chrysantmeum seedlings, we carried out histochemical staining experiments on the leaves. As a whole, chrysanthemum leaves were subjected to more severe oxidative stress under the conditions of freezing than chilling; and under freezing conditions, seedling with prior CA (T03) accumulated less H_2_O_2_ and O_2_^−^ than seedlings without CA (T04) (Fig. 5b). Furthermore, we determined activities of antioxidant enzymes (POD and CAT) in chrysanthemum. Under chilling stress, POD activity of seedlings was not significantly different than normal condition, but CAT activity increased obviously. Under freezing conditions, both POD and CAT activities significantly increased, and activities of T03 were remarkably higher than T04 (Fig. 5d, e).

Moreover, we measured the changes in the osmoregulation substances of chrysanthemum at low temperatures. Under chilling stress, both proline and soluble sugar contents of chrysanthemum were increased. And under freezing condition, proline and soluble sugar contents of T03 were substantially increased, whereas T04 was low in proline and soluble sugar contents (Fig. 5f). The result indicated that CA can induce chrysanthemum osmotic regulation systems to work better under freezing conditions.

## Discussion

Low temperature is an important stress factor for plants. It can not only limit the widespread distribution of plants, but also seriously affect the growth and development of them, and cause a series of adverse metabolic reactions [9]. However, ‘jinba’ exactly is a kind of cutting-chrysanthemum which is difficult to mass-produce in winter with normal form. And now, the data available on the molecular basis of chrysanthemum response to cold stress is not limited any longer with the development of high-throughput sequencing.

Based on the quantitative statistics of DEGs in each comparison, we found a lot of genes induced by low temperature and the majority of them involved in up-regulation rather than down-regulation (Fig. 2b). From Fig. 2a, we found that some genes may only be induced by freezing and function on protecting chrysanthemum from freezing damage. As expected, more genes were up-regulated in T03 than T04 (Fig. 2b), supporting the statement that CA process may contribute to plants obtaining increased tolerance to freezing conditions [20].

### KEGG analysis

As shown in Table 6, when exposed to chilling conditions, DEGs of chrysanthemum associated with biosynthesis and metabolism pathways were enriched more than freezing conditions, suggesting that chilling stress could induce more biosynthesis and metabolism genes than freezing. However, the pathways of ‘Plant-pathogen interaction’ and ‘Plant hormone signal transduction’ were greater enriched in freezing condition than chilling. Plant-pathogen interaction pathway involves lots of sensing and signaling genes related to Ca^2+^ signal transducion pathway and MAPK cascade, it demonstrated that more genes participated in the signal sensing and transduction pathways were induced by freezing than chilling stress.

In addition, under freezing stress, whether biosynthesis and metabolism related pathways or signal related pathways in CP2 were greater enriched than CP3. It indicated that CA can relieve the inhibition of biosynthesis and metabolism by freezing injury, and strengthen the response of signal transduction pathway to freezing injury.

### Low temperature signal transduction

For surviving in low temperature environment, plants developed a series of complex signaling pathways, and the activities of which are modulated by adding or removing phosphate groups [21]. Lots of protein kinases and protein phosphatases participate in the processes of phosphorylation and de-phosphorylation, such as RLKs, MAPKs, CDPKs, CIPKs, protein phosphatase P (PPP), protein phosphatase M (PPM), and protein tyrosine phosphatase (PTP) [22]. Larges of previous experiments implicated that RLKs work on hormone signaling events in response to environmental stimuli [23–26]. In our experiment, lots of RLKs were found both in all treatments, and up-regulated RLKs in freezing stress were more than in chilling stress (Additional file 2: Table S1). The MAPK cascade activated by reactive oxygen species mediates cold stress signaling in *Arabidopsis* [27]. Plants over-expressing *MKK2* increased freezing tolerance, whereas *mkk2* null mutant plants were hypersensitive to cold stress [27]. Overexpression of *OsMAPK5* conferred cold tolerance in rice seedlings [28]. We respectively identified 2, 8, 10 MAPKs genes up-regulated in CP1, CP2 and CP3, suggesting that MAPK cascade played a significant role in the low temperature stress, especially in freezing stress in chrysanthemum. In addition, protein phosphatase has been shown to be crucial components of MAPKs cascade [21]. Relevantly, several PPP, PPM and PTP differentially expressed in each treatment were identified. CDPKs and CIPKs are two key kinases of the Ca^2+^-signaling pathway, which is known to be involved in responses to cold stimuli [29, 30]. And it has been reported that *OsCIPK03, OsCIPK12, OsCDPK7* and *OsCDPK13* played important roles in conferring cold tolerance in rice [31–33]. In this study, 3, 12, 9 CDPK and 7, 5, 4 CIPK genes were differentially expressed in three treatments, respectively, and all of them were up-regulated. These genes may provide excellent genetic resources for breeding engineering of low-temperature tolerance chrysanthemum.

The different expression of genes involving in the signal cascade mechanism can affect the expression of genes participating in the formation of plant hormones such as ABA, ET, SA and JA. And then, theses hormones may amplify the cascades or initiate some new signaling pathways [34]. In this experiment, ABA, ET and JA are top three responsive hormone signal transduction pathways in chrysanthemum, and there were numbers of DEGs involved in them. ABA is a plant hormone extensively involved in environmental stresses. ABA-responsive element (ABRE) and dehydration-responsive element (DRE) were two cis-acting elements involved in ABA-mediated gene expression [21]. Besides DEGs encoding three key proteins (ABA receptor, PP2C and SnRK2) of ABA signaling pathway were identified, another one ABF and 11 DRE-binding factors (DREB) were obtained in our study (Additional file 3: Table S2). Both ABF and DREB can activate the down-stream genes expression in response to cold stress [35]. This indicated that low temperature-induced expression of chrysanthemum appeared to be ABA dependent.

Lipid molecules are also involved in signal transduction during low temperature stress. Phosphatidic acid (one kind of membranous secondary messenger molecule) can be produced by phospholipase D (PLD) [36]. Li et al. have shown that the cold-induced freezing tolerance of *Atpldd* T-DNA knock-out mutant *Arabidopsis* was impaired, whereas that of *AtPLDd*-overexpression plants was enhanced [37]. In our study, we totally found 6 PLD DEGs, 3, 4 and 2 of which represented up-regulation in CP1, CP2 and CP3, respectively. The up-regulation amplitude of PLD genes in CP2 was higher than CP3. This indicated that PLD was closely related to low-temperature tolerance of chrysanthemum, and CA might contribute to improve freezing tolerance of chrysanthemum.

### TFs responding to low temperature

When subjected to low temperature stress, TFs could be activated through a series of signal transduction pathways. Then activated TFs bind specifically to the corresponding cis-acting element to activate the expressions of various downstream resistance-related genes, thereby enhancing the cold tolerance of plants [38]. AP2/ERF, WRKY, MYB-related, MYB, NAC were five major TF classes identified as DEGs in response to low temperature stress. And some members of above TF families have been identified as regulators involved in low temperature stress responses [39–41]. Among various reported TFs, DREBs/CBFs played pivotal roles in improving low temperature tolerance of plants [34]. Overexpression of *Arabidopsis DREBs* enhanced chilling or freezing tolerance in many plant species, and overexpression *DREB1* of several plant species in transgenic *Arabidopsis* also conferred tolerance to freezing stress [34]. In our study, a total of 10 DREBs were up-regulated under chilling or freezing stress. Interestingly, there were only 3 WRKY and 3 MYB TFs up-regulated in the chilling treatment, whereas respectively about 17 were up-regulated in freezing conditions. It can be inferred that WRKY and MYB TFs may play vital roles in response to the freezing stress. Moreover, some other TF families were also found to be differentially expressed in our study, such as bHLH, bZIP, C2H2, GRAS and C3H. These results suggested that a large number of TFs were involved in the regulation mechanism of chrysanthemum in response to low temperature stress through different pathways.

### Cold resistant genes associated with the biofilm system

When the temperature drops, phase changes firstly occurs in the biofilm membrane lipid, which leads to the increase of membrane permeability and the leakage of electrolyte. Physiologically, Fig. 5c showed the chrysanthemum biofilm system suffered more severe persecution under freezing conditions, especially T04 chrysanthemum. Therefore, we explored the molecular mechanisms of biological membranes. Increasing the proportion of unsaturated fatty acids in membrane lipids is propitious to membrane fluidity, thus decreasing the phase changes temperature of the membrane lipids [42]. Therefore, the content of membrane fatty acid and the fluidity of membrane are closely related to the cold resistance of plants. In molecular terms, we found a total of 12 genes encoding LTPs and 7 genes encoding FADs were differentially expressed from three libraries (CP1, CP2, and CP3). Among them, 7 FADs genes included omega-6 and omega-3 fatty acid desaturases genes, which are mainly involved in processes of unsaturated fatty acids (linoleic acid and α-linolenic acid) biosynthesis. And previous reports showed that increasing omega-3 desaturase genes expression could enhanced resistance of different plants to cold stress [43, 44]. Meanwhile, PLD and secretory phospholipase A2 (SPLA2) were also two favorable factors of membrane fluidity [45, 46]. Relevantly, we found numbers of DEGs encoding PLD and SPLA2 in three libraries. These results indicated that chrysanthemum may be able to tolerant low temperatures by enhancing the stabilization of cell membrane. In general, genes encoding LTPs, FADs, PLDs and SPLA2 were obviously much more active in chrysanthemum with CA (T03) than in chrysanthemum without CA (T04) (Table 7), suggesting that chrysanthemum acquired freezing tolerance from CA process.

The damage of cell membrane system under low temperature stress may be related to membrane lipid peroxidation induced by free radical and ROS. Membrane lipid peroxidation product malondialdehyde (MDA) conbined with protein can cause membrane protein denaturation, so membrane lipid peroxidation is also an important reason leading to the decrease of membrane lipid fluidity [47]. Fig. 5b showed that chrysanthemum was subjected to more severe reactive oxygen injury under freezing stress than chilling stress, and T04 was more serious than T03. The increase of contents or activities of protective enzymes is beneficial to maintain the balance between ROS production and removal. Under the same freezing condition, activities of POD and CAT enzymes of T03 was higher than that of T04, and more genes encoding peroxidase represented up-regulated in T03, suggesting that CA process induced chrysanthemum to establish a better protective enzyme system to improve their ability to resist frost damage.

In addition, under low temperature stress, the genes encoding antifreeze proteins and molecular chaperones altered to provide protection to plants. LEAs are known for its aggressive response to dehydration; however, its function in freezing tolerance has been confirmed [48]. A total of 16 LEA protein genes among DEGs were identified in our study. Some cold-responsive genes encoded molecular chaperones like HSP70s and HSP90s. A *Brassica napus* gene *hsp90* contributed to freezing tolerance by stabilizing proteins against freeze-induced denaturation [49]. There were 9, 21 and 14 genes encoding HSPs identified in DEGs of CP1, CP2 and CP3, speculating that HSPs chaperones played important roles in resistance to low temperature stresses in chrysanthemum.

### Cold resistant genes associated with osmotic regulation system

Osmotic adjustment is a regulation of plants when dehydration stress evocked by low temperature occurs. As shown in Fig. 5g, low temperature induced the accumulation of soluble sugar in chrysanthemum. Soluble sugar, as an osmotic regulator and an anti dehydrating agent, can reduce cell water potential and enhance water holding capacity [50]. Sugar can provide carbon source and substrate to induce other related cold resistance and physiological and biochemical process, contributing to the enhancement of cold resistance; it also can avoid the protein coagulating caused by the low temperature, to further improve the cold resistance of plant [51, 52]. Significant changes in expressions of the key enzyme genes involved in starch and cellulose degrading, soluble sugars synthesis, and galactose degradation. α−/β-amylase and β-glucosidase were respectively involved in the degradation of starch and cellulose, and the genes encoding these enzymes showed different degrees of rise. Meanwhile, the genes involved in the processes of sucrose, trehalose and raffinose synthesis were also up regulated, which encoding sucrose-phosphate synthase, sucrose synthase, trehalose phosphate synthase, trehalose phosphatase, galactinol synthase, and raffinose synthase. The previous study reported that overexpression of sucrose-phosphate synthase promoted sucrose levels and increased the degree of cold tolerance in transgenic Arabidopsis [53]. What’s more, we found aldose 1-epimerase (galM), which acts on the degradation of galactose, and alpha-galactosidase (galA), which hydrolyze substances containing galactoside bonds, were down regulated in T02 or T03. It’s beneficial to maintain the stability of soluble sugar content.

Low temperature also induced the accumulation of proline in chrysanthemum (Fig. 5f). When plants are subjected to cold stress, proline can not only reduce cell water potential, but also promote protein hydration, thus playing a certain role in cells protection [54]. P5CS, which participates in the synthesis of proline, was only found to be up-regulated in T02 and T03. PRODH, which participates in the degradation of poline, was only represented up-regulated in T04. It suggests that T03 has a better capacity of maintaining high levels of proline than T04, which is one of reasons why T03 is more resistant to freezing than T04.

## Conclusion

Our study presents a genome-wide transcript profile of *Dendranthema grandiflorum* var. jinba, which supported by physiological tests, and provides insights into the molecular mechanisms of *D. grandiflorum* in response to low temperature. Analyses of physiological and molecular data showed that CA could lead to the establishment of cold resistance mechanism of chrysanthemum, and finally improve the adaptability of chrysanthemum to freezing. And we explored a series of potential chilling-resistant and antifreeze genes associated with low temperature signal transduction, biofilm system and osmotic regulation system, which can serve as candidate genes for cold-tolerance molecular breeding project of chrysanthemum.

## Additional files

Additional file 1: **Figure S1.** Composition of raw reads in the four RNA libraries. **Figure S2.** Functional classification and pathway assignment of DEGs by KEGG. The y-axis indicates the name of the KEGG pathways. The x-axis indicates the percentage of the number of annotated DEGs under the pathway in total number of DEGs in all pathways. **Figure S3.** Transcription factor families occupied proportion in *Dendranthema grandiflorum* DEGs. (DOC 1620 kb)

Additional file 2: **Table S1.** Differentially expressed protein kinases and protein phosphatases genes involved in low temperature responses. (XLS 74 kb)

Additional file 3: **Table S2.** Genes involved in ABA signaling pathway and DREBs in response to low temperature. (XLS 27 kb)

## Abbreviations

ABA: Abscisic acid
ABF: ABA-responsive element binding factor
ABRE: ABA-responsive element
AFPs: Anti-freezing proteins
BP: Biological Processes
BR: Brassinosteroid
CA: Cold acclimation
CAT: Catalase
CC: Cell Component
CDPK: Calcium-dependent protein kinase
CIPK: CBL-interacting protein kinase
CK: Cytokinin
COG: Cluster of orthologous groups
COR: Cold-regulated gene
DEGs: Differentially expressed genes
DRE: Dehydration-responsive element
DREB: DRE-binding factors
EC: Electrolyte conductivity
eggNOG: Evolutionary genealogy of genes: Non-supervised Orthologous Groups
ET: Ethylelne
FADs: Fatty acid desaturase
FPKM: Fragments per kilobase of transcript per million fragments mapped
GA: Gibberellin
galA: Alpha-galactosidase
galM: Aldose 1-epimerase
GLPs: Beta-1,3-glucanase proteins
GO: Gene Ontology
HSPs: Heat shock proteins
JA: Jasmonic acid
KEGG: Kyoto Encyclopedia of Genes and Genomes
KOG: euKaryotic Orthologous Groups
LEAs: Late-embryogenesis-abundant proteins
LTPs: Lipid-transfer proteins
MAPK: Mitogen-activated protein kinase
MDA: Malondialdehyde
MF: Molecular Function
NGS: Next-generation sequencing
Nr: NCBI non-redundant
P5CS: Pyrroline-5-carboxylate synthetase
Pfam: Protein family
PGIPs: Polygalacturonase-inhibiting proteins
PK: Protein kinase
PLD: Phospholipase D
POD: Peroxidase
PP: Protein phosphatase
PP2C: Protein phosphatase 2C
PPM: Protein phosphatase M
PPP: Protein phosphatase P
PTP: Protein tyrosine phosphatase
qRT-PCR: Quantitative real-time polymerase chain reaction
RLKs: Receptor-like kinases
ROS: Reactive oxygen species
RSEM: RNA-Seq by Expectation Maximization
SA: Salicylic acid
SnRK2: Serine/threonine-protein kinase
SPLA2: Phospholipase A2
SPS: Sucrose-phosphate synthase
TFs: Transcription factors
TLPs: Thaumatin-like proteins
TPP: Trehalose phosphatase
TPS: Trehalose phosphate synthase
