# Supplementary material for: Transcriptome analysis of chrysanthemum (Dendranthema grandiflorum) in response to low temperature stress
Source: BMC Genomics. 2018 May 2;19:319. doi: 10.1186/s12864-018-4706-x (PMC5930780; doi:10.1186/s12864-018-4706-x)
Supplement: Supplementary file 1 — Figure S1. Composition of raw reads in the four RNA libraries. Figure S2. Functional classification and pathway assignment of DEGs by KEGG. The y-axis indicates the name of the KEGG pathways. The x-axis indicates the percentage of the number of annotated DEGs under the pathway in total number of DEGs in all pathways. Figure S3. Transcription factor families occupied proportion in Dendranthema grandiflorum DEGs. (DOC 1620 kb) [file 12864_2018_4706_MOESM1_ESM.doc]

**Transcriptome analysis of chrysanthemum (*****Dendranthema grandiflorum*) in response to low temperature stress**

Ke Wang, Zhen-yu Bai, Qian-yu Liang, Qing-lin Liu*, Lei Zhang, Yuan-zhi Pan, Guang-li Liu

Department of Ornamental Horticulture, Sichuan Agricultural University, 211 Huimin Road, Wenjiang District, Chengdu, Sichuan 611130, P.R. China

*Corresponding author; E-mail: qinglinliu@126.com;

Tel/Fax: +86-28-86290881.

**Additional file 1**

**

**

**Figure S1. Composition of raw reads in the four RNA libraries.**

**

**

**

**

**Figure S2. Functional classification and pathway assignment of DEGs by KEGG.** The y-axis indicates the name of the KEGG pathways. The x-axis indicates the percentage of the number of annotated DEGs under the pathway in total number of DEGs in all pathways.

**
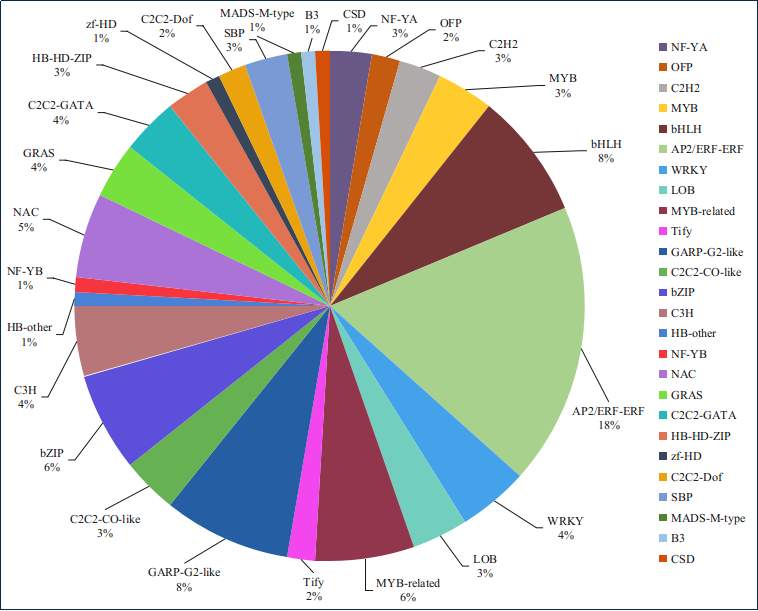
**

**CP1**


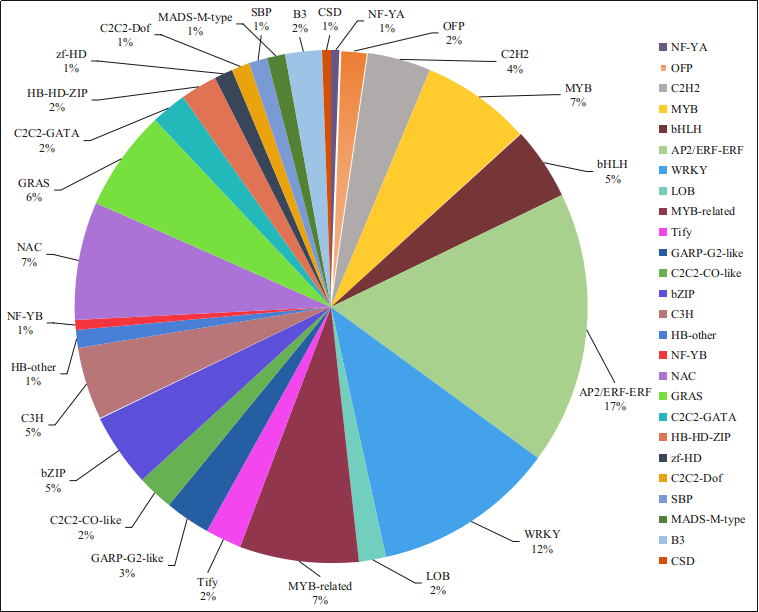
**CP2**

**
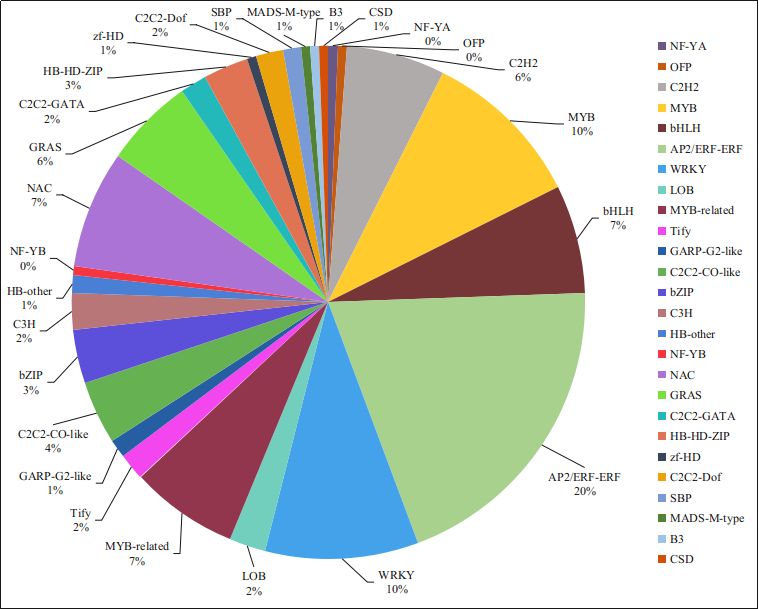
**

**CP3**

**Figure S3. Transcription factor families occupied proportion in *Dendranthema grandiflorum* DEGs*.***
